# Persisting and Increasing Neutrophil Infiltration Associates with Gastric Carcinogenesis and E-cadherin Downregulation

**DOI:** 10.1038/srep29762

**Published:** 2016-07-14

**Authors:** Hualin Fu, Yue Ma, Meng Yang, Chunlei Zhang, Hai Huang, Ying Xia, Lungen Lu, Weilin Jin, Daxiang Cui

**Affiliations:** 1 Institute of Nano Biomedicine and Engineering, Key Laboratory for Thin Film and Microfabrication of the Ministry of Education, Department of Instrument Science and Engineering, School of Electronic Information and Electrical Engineering, Shanghai Jiao Tong University, 800 Dongchuan Road, Shanghai, 200240, China; 2 National Center for Translational Medicine, Shanghai Jiao Tong University, 800 Dongchuan Road, Shanghai, 200240, China; 3 Department of Clinical Biochemistry, School of Clinical Laboratory Science, Guizhou Medical University, Guiyang, Guizhou 550004, China; 4 Department of Gastroenterology, Shanghai First People’s Hospital of Shanghai Jiao Tong University School of Medicine, Shanghai 200240, China

## Abstract

H. pylori-induced chronic inflammation is considered the most important cause of gastric cancer. The actual process how chronic inflammation triggers gastric carcinogenesis is still not clear. In this study, neutrophils and relative markers in gastric cancer development were examined with immunohistochemistry and fluorescence RNA *in situ* hybridization methods. On average, 24 times more neutrophils were found in gastric cancer tissues and about 9 times more neutrophils were found in gastric intestinal metaplasia tissues comparing to normal gastric tissue controls. CagA^+^ H. pylori infection in cancer adjacent tissues or EBV infection in cancer tissues did not increase neutrophil infiltration into gastric cancer tissues significantly. Neutrophil density was positively correlated with cell proliferation while negatively correlated with E-cadherin intensity. E-cadherin is also transcriptionally downregulated in gastric cancer tissues comparing to adjacent tissue controls. The increased neutrophils in the gastric cancer tissues appear to be related to increased chemoattractant IL-8 levels. In gastric cancers, neutrophil numbers were higher comparing to cancer adjacent tissues and not associated with patient ages, tumor invasion depth, tumor staging, metastasis or cancer types. The conclusion is that persisting and increasing neutrophil infiltration is associated with E-cadherin downregulation, cell proliferation and gastric carcinogenesis.

Tumorigenesis is an inflammation-driven process^1^,^2^. Over 15% of human cancers are linked to inflammations induced by known infectious agents. Since inflammation is not only induced by pathogen infections, but also can be triggered in autoimmune disease or by stress under sterile conditions called “sterile inflammation”, the link between inflammation and cancer might be even stronger than previously thought^3^,^4^. Tumor stromal cells and infiltrating leukocytes such as lymphocytes, macrophages, mast cells, NK cells, dendritic cells and neutrophils, form the “other half” of tumor tissue cell compartments, which might play both tumor inhibition or tumor promotion roles. Macrophages are well-known to inhibit or promote tumorigenesis by adopting different cell fates, namely M1 macrophages and M2 macrophages^5^. Neutrophils, accounting to 50–70% of leukocytes, were once thought to play negligible role in cancer development because of their short life-span and differentiated phenotype^6^. Comparing to neutrophils, macrophages/monocytes comprise of 3–8% leukocytes, while the studies of tumor associated macrophages is roughly 10 times more than the studies of tumor-associated neutrophils in the current Pubmed database. The studies of tumor associated neutrophils are likely under-represented. Nevertheless, research progress on neutrophils over the last ten years, with discoveries of “neutrophil extracellular trap formation” and “N2 neutrophils”, indicated that neutrophils might be active players in cancer development^7^,^8^. Several published studies emphasized the role of neutrophils functioning as immunosuppressive cells in the cancer environment^9^,^10^,^11^,^12^. Elevated neutrophil to lymphocyte ratio in the peripheral blood is considered as an important prognostic marker for poor survival in a variety of cancers including lung cancer, colon cancer, breast cancer and gastric cancer^13^,^14^,^15^,^16^,^17^. However, the exact mechanism for the elevation of neutrophil to lymphocyte ratio in the peripheral blood in cancer patients is not clear.

Gastric cancer is one of the prototypical cancers that triggered by chronic inflammation. Infection by H. pylori is considered the most important causes of gastric cancer development^18^,^19^. Whether neutrophils are involved in gastric cancer development is not very clear. However, a number of previous studies suggested that neutrophils might be important markers for gastric cancer prognosis^17^,^20^,^21^. Several histological studies found that 7.6% to 15.5% of gastric carcinoma is enriched for neutrophil infiltrations^22^,^23^. However, there are conflicting reports regarding whether tissue neutrophil infiltration is associated with lymph node metastasis or tumor staging^20^,^23^. Most of these studies focused on the relation of neutrophils and gastric cancer prognosis, whether neutrophil infiltration also contributes to gastric cancer initiation and early development is not investigated.

In the present study, we used antibody immunofluorescence staining to identify neutrophils with high specificity and high resolution in gastric cancer and gastritis tissue arrays. We studied neutrophil infiltration patterns during the early steps of gastric cancer development process, from gastritis, gastric intestinal metaplasia to gastric cancer. We also studied the neutrophil distributions in the tumor adjacent tissues and normal gastric tissues and established the basal levels of neutrophils. Additionally, H. pylori infection and EBV infection was studied in the context of neutrophil infiltration. The effect of neutrophil infiltration on gastric epithelium cell proliferation and E-cadherin protein expression was examined. The transcriptional regulation of E-cadherin, the neutrophil marker CD11b and the neutrophil chemoattractant molecule IL-8 in gastric cancers was also investigated with RT-PCR.

## Results

### Immunohistochemical analysis of neutrophils on gastritis and gastric cancer tissue sections

**Figure 1:**
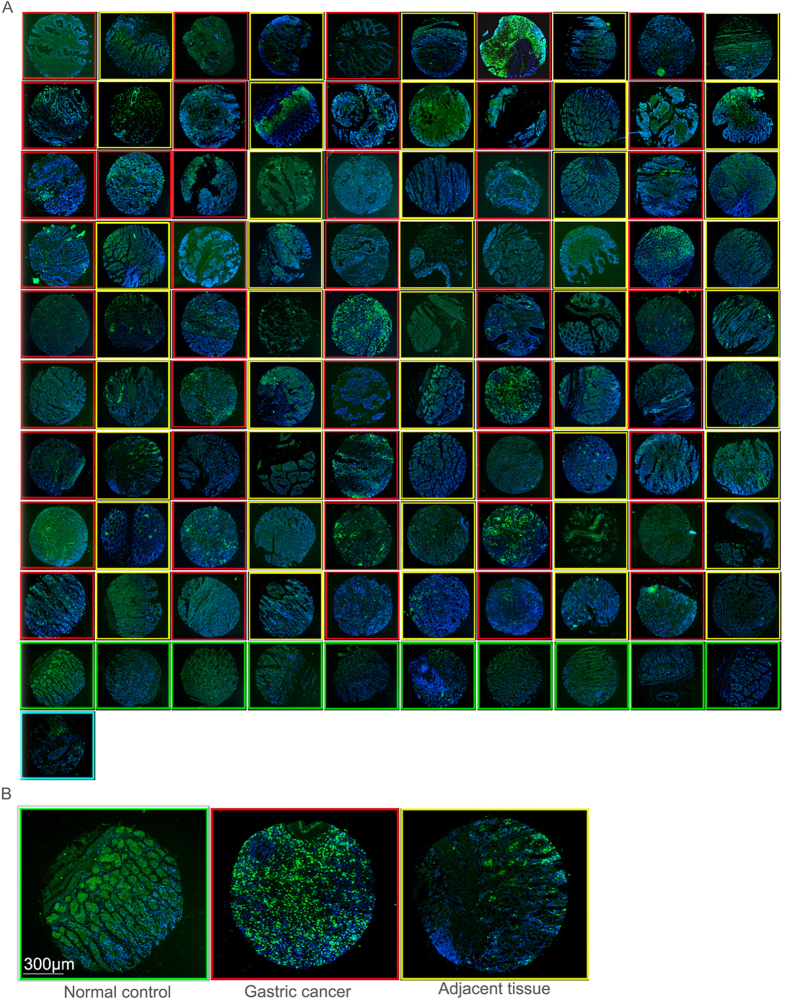
Neutrophil marker MPO expression in gastric cancer and adjacent tissue and control tissues. (**A**) Overview of MPO neutrophil staining in the gastric cancer versus cancer adjacent tissue array. For the first 9 rows, each row has 5 pair samples. Each pair of samples consists of one cancer sample (with a red fame) and one cancer adjacent sample (with a yellow frame) with the cancer sample comes first (with exception that the first pair of samples of line 3 happened to be both cancerous tissues). The last 11 samples contains 10 normal gastric tissue samples (with green frames) (although one of them still has lymphocyte infiltration that was excluded for analysis) and one tissue sample of liver cancer (with a light-green frame). Scale bar, 300 μm (**B**) Representative magnified pictures of control, gastric cancer and cancer adjacent tissue samples Scale bar, 300 μm. Positive experiments to show that MPO is a neutrophil specific marker could be seen in Supplementary Fig. 1.

**Figure 2:**
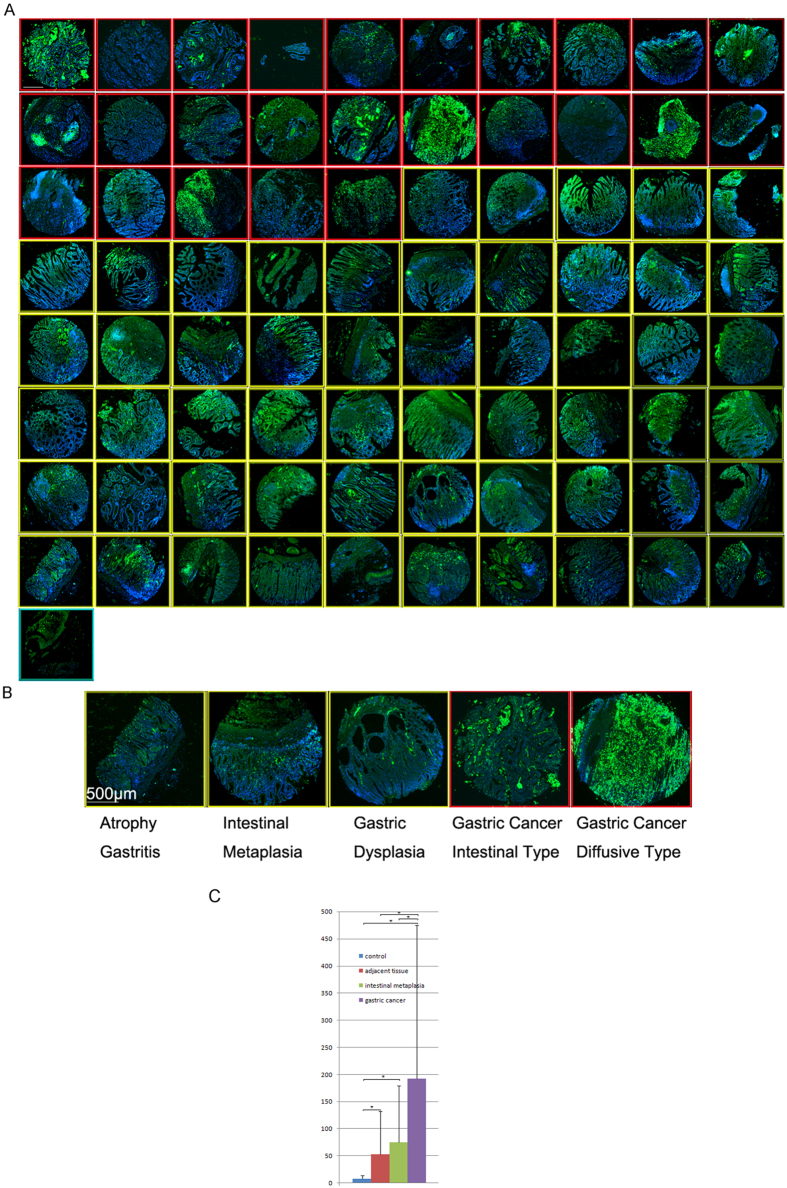
Neutrophil marker MPO expression in gastric cancer and gastric intestinal metaplasia tissues. (**A**) Overview of the gastric cancer and intestinal metaplasia tissue MPO array. The first 25 samples are gastric cancer samples (with red frames) while the rest 55 are gastritis samples (with yellow frames) including 38 gastritis samples with intestinal metaplasia. The last sample is a liver cancer tissue marker. Scale bar, 500 μm. (**B**) Representative magnified pictures of neutrophils in atrophy gastritis, gastric intestinal metaplasia, gastric dysplasia (yellow frames), and gastric cancer samples (red frames). Scale bar, 500 μm. (**C**) Neutrophil counting results of different type of tissue samples. There is a significant increase of neutrophil numbers in gastric cancer tissues comparing to normal controls (p = 0.0001), adjacent tissues (p < 0.0001), intestinal metaplasia tissues (p = 0.02). There is also significant increase of neutrophils in cancer adjacent tissues (p = 0.001) and intestinal metaplasia tissues (p = 0.0001) comparing to normal controls. *Indicated the difference between the two groups is statistically significant.

Neutrophil marker MPO immunostaining of neutrophils was carried out on a gastric cancer, gastric cancer adjacent tissue array (Array 1) (Fig. 1A) and a gastric cancer and gastritis tissue array (Array 2) (Fig. 2A). To increase sample numbers, MPO immunohistochemistry was also carried out on a third array with more gastric cancer and cancer adjacent tissues (Array 3) (The data analysis from this array was included in Fig. 2C and Table 1). The evidence to show that MPO is indeed a neutrophil-specific marker is provided in Supplementary Fig. 1. In total, the neutrophil counting analysis included 138 gastric cancer samples, 116 cancer adjacent tissues, 50 gastritis samples and 9 normal tissue controls. Neutrophil numbers in control normal gastric tissues are by average 8 ± 6 on a 1 mm-diameter tissue spot with a section thickness of 5 μm (Figs 1 and 2C). Neutrophil numbers in gastric cancer adjacent tissues (54 ± 79) is roughly 6.8 times higher than the neutrophil number in normal controls (p = 0.001). Neutrophil numbers in gastric cancer tissues (193 ± 284) are about 3.6 times higher than gastric cancer adjacent tissues (p < 0.0001)(Figs 1 and 2C). Compare to normal gastric tissues, neutrophil number in gastric cancer tissues is 24 times higher (p = 0.0001) (Figs 1 and 2C). Although many of the cancer adjacent tissues were identified or even labeled by the tissue array vendors as gastritis tissues after histological examinations, however after analyzing all of the adjacent tissue samples, the average neutrophil numbers in cancer adjacent tissues (54 ± 79) is slightly smaller than gastritis tissues (66 ± 62) (p = 0.02). As expected, neutrophil numbers in gastritis tissues are also significantly larger than normal tissue controls (p < 0.0001).

Neutrophil infiltration into gastritis tissues with intestinal metaplasia was analyzed further. In total, neutrophil numbers in 39 intestinal metaplasia tissue samples were counted. Significantly increased neutrophil infiltration (roughly 9 times of increase) was found in intestinal metaplasia samples comparing to normal controls (77 ± 106 vs. 8 ± 6, p = 0.0001, Fig. 2C) (Intestinal metaplasia samples found in the cancer adjacent tissues were also included). However, neutrophil numbers in intestinal metaplasia samples were significantly smaller than in gastric cancer tissues (77 ± 106 vs. 193 ± 284, p = 0.02). Gastritis samples without intestinal metaplasia had mean neutrophil numbers of 64 ± 55, which is not significantly different from neutrophil numbers in gastritis samples with intestinal metaplasia (p = 0.90), however, still significantly higher than in normal gastric tissue controls (p = 0.0003). Representative pictures of neutrophil infiltration of gastric tissues at different stages of gastric cancer development, such as atrophy gastritis, gastric intestinal metaplasia, dysplasia, intestinal type of gastric cancer and diffusive type of gastric cancer were shown in Fig. 2B.

### Analysis on the relation of H. pylori and EBV infection with neutrophil infiltration into gastric tissues

**Figure 3:**
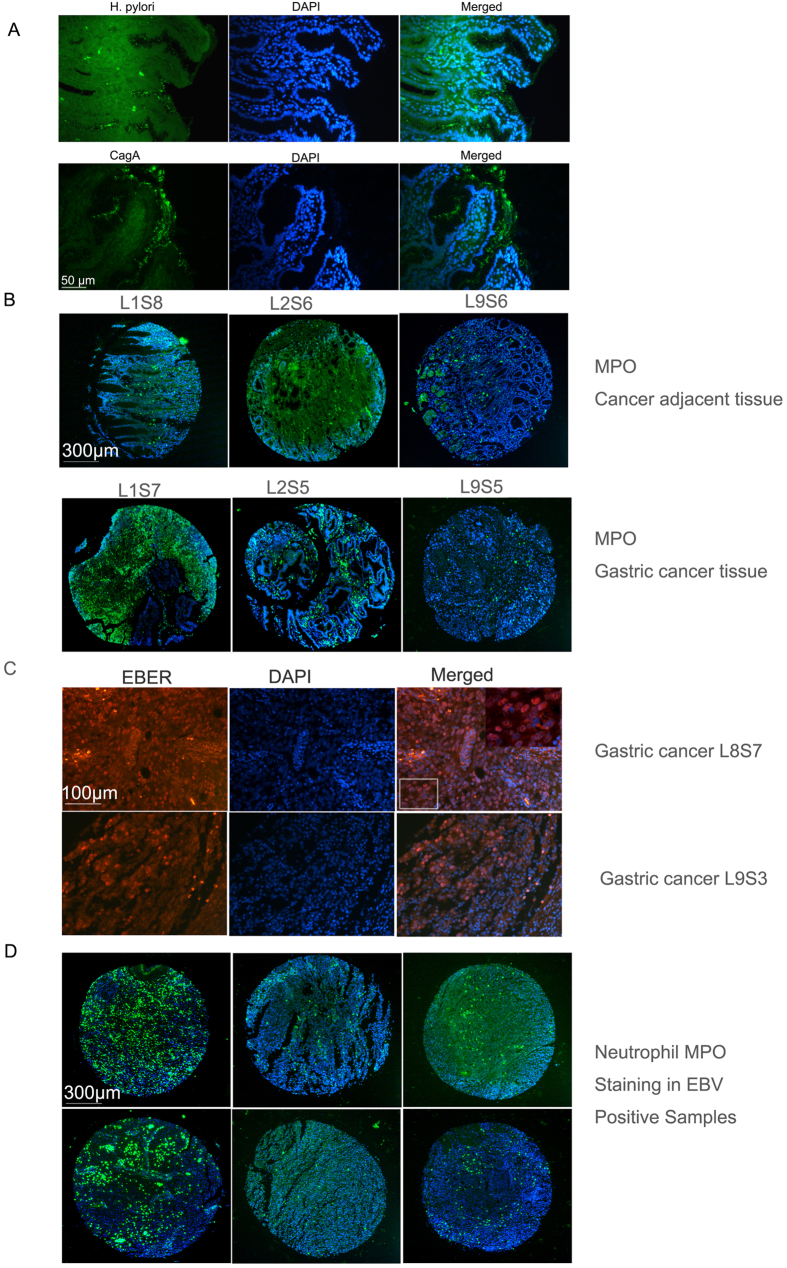
Relation of Neutrophil infiltration and H pylori infection and EBV infection. (**A**) Images of H. pylori infection showed by a general H. pylori antibody and the H. pylori CagA antibody staining. Scale Bar, 50 μm. (**B**) Neutrophil MPO staining in 3 pairs of cancer adjacent tissues (positive for H. pylori) and corresponding cancer tissues. Scale Bar, 300 μm. (**C**) Two images of gastric cancer samples positive for EBV infection. Magnified images showed the nuclear localized EBER1/2 *in situ* hybridization signals. Scale Bar, 100 μm. (**D**) Neutrophil MPO staining of 6 EBV positive gastric cancer samples. Scale Bar, 300 μm. Positive control experiments for CagA antibody and EBER *in situ* could be found in Supplementary Fig. 2 and Fig. 3.

We also checked the H. pylori infection status on Array 1 and Array3 by immunostaining of H. pylori CagA protein. Since H. Pylori CagA positive rate is reported from 70% to over 90% of gastric patients both in China and in US, we think CagA could be a useful marker for H. pylori infections^24^,^25^,^26^,^27^,^28^. In addition, since CagA positive strain is more cytotoxic than CagA negative strains^29^, using CagA as the H. Pylori marker probably is more relevant to gastric diseases. CagA antibody staining showed similar but broader staining pattern at gastric mucosa cell membranes as a general H. pylori antibody (Fig. 3A). The reason CagA antibody showed broader staining at cell membranes could be due to the well-known phenomenon of CagA translocation at the cell membrane upon H. pylori infection^30^,^31^,^32^. Additional positive control experiment for the CagA antibody was provided in Supplementary Fig. 2. CagA^+^ H. pylori infection was found in the tumor adjacent tissues but not cancer tissues. 21 cancer adjacent tissue samples had prominent CagA^+^ H. pylori infection. The average number of neutrophil infiltration in CagA^+^ H. pylori positive adjacent tissues is significantly higher than CagA^+^ H. pylori negative adjacent tissues in these two tested arrays (87 ± 88 vs. 47 ± 76, p = 0.0005), confirming that H. pylori infection attracts neutrophil infiltration into gastritis tissues as previously reported^33^,^34^. However, neutrophil numbers of the cancer samples with CagA^+^ adjacent tissues is not statistically different from the neutrophil numbers of the cancer samples with CagA negative adjacent tissues (245 ± 367 vs. 185 ± 270, p = 0.19). Representative pictures of CagA^+^ H. pylori infections and neutrophil marker MPO expression in CagA^+^ H. pylori positive adjacent tissues and corresponding cancer tissues are shown in Fig. 3A,B.

To investigate the possible influence of EBV infection on neutrophil influx, we did EBER *in situ* hybridization on Array 1 to detect EBV infections. Positive control experiments of EBER *in situ* hybridization were shown in Supplementary Fig. 3. We found 6 samples in Array 1 (L6S7, L7S9, L8S1, L8S7, L9S3, L9S7. L: Line; S: Sample) were EBV-infected but none of the tumor adjacent tissue samples were infected, suggesting gastric cancer cells are preferentially infected by EBV (EBV EBER *in situ* results were shown in Fig. 3C). Among these samples, 4 of them are intestinal-type of gastric cancers while 2 of them are diffusive type of gastric cancers. The ratio of positive EBV infection in gastric cancer (13%) is similar to the data reported by previous studies^35^. The average number of neutrophil influx of these EBV-positive samples is 397 ± 492, although higher than the average number in the rest of cancer samples of this tissue array (225 ± 387), the difference is not statistically significant (p = 0.21) (Fig. 3D). These data pointed out that EBV infection does not lead to statistically significantly higher neutrophil infiltration into gastric cancer tissues comparing to EBV-negative cancer tissues.

### Analysis of neutrophil distribution patterns in different gastric tissues

**Figure 4:**
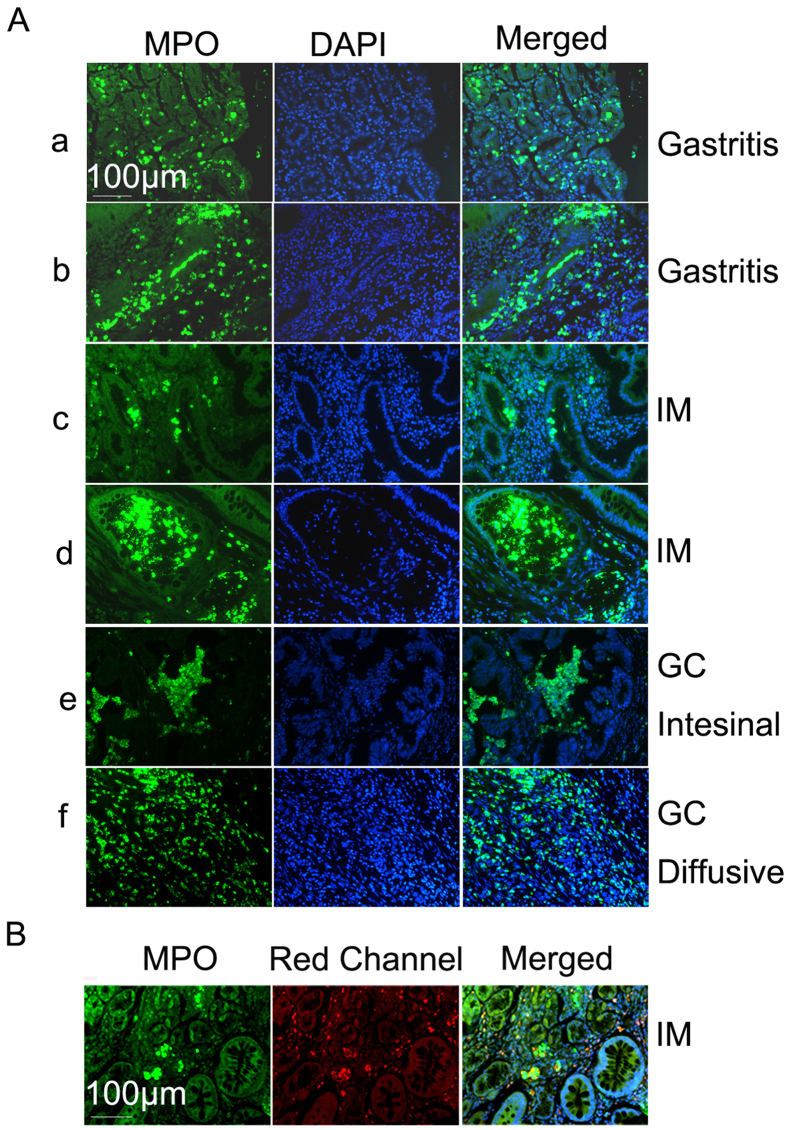
Distribution patterns of neutrophils in gastritis, intestinal metaplasia and gastric cancer tissues. (**A**) Distribution patterns of neutrophils showed neutrophils in the stroma tissues, crossing epithelium layer and infiltrating into the lumens of gastric glandular epithelium (a,b), intestinal metaplasia epithelium (c,d), and the center of gastric cancer tissues (e). In diffusive gastric cancers, neutrophils were often evenly dispersed and intermingled with cancer cells (f). (**B**)Neutrophils are also found enriched in blood vessels within gastric intestinal metaplasia tissues. IM, intestinal metaplasia. GC: gastric cancer. Scale bar, 100 μm.

Detail analysis on the pattern of neutrophil infiltration showed that neutrophils are penetrating through tissue stroma, epithelium and entering the glandular cavities formed by the epithelium tissues. In gastritis tissues, neutrophils could be observed in the gastric stroma, epithelium layers and also in the center cavities of gastric glands (Fig. 4A(a,b)). In intestinal metaplasia tissues, neutrophils could be observed crossing intestinal metaplasia epithelium layers and could also be observed in the center of metaplastic glands (Fig. 4A(c,d). In gastric cancer tissues, neutrophils could be also observed in the central cavity formed by gastric cancer epithelium (Fig. 4A(e)). In all three different tissue types, neutrophils are seen crossing the epithelium layers and migrating into the center of the tissues, suggesting certain signaling gradients are driving neutrophil migration into the center of these different tissues similarly. Moreover, the patterns of neutrophil distribution seemed to be different in different gastric cancer types. Neutrophil aggregations were usually segregated with the gastric cancer cells in intestinal type of gastric cancers (Fig. 4A(e)). However, in diffusive gastric cancer tissues, neutrophils showed an evenly distributed intermingled pattern related to gastric cancer cells, suggesting a different interaction pattern of neutrophils and gastric cancer cells comparing to that in intestinal type of gastric cancers (Fig. 4A(f)). However, the numbers of neutrophils in diffusive cancers (216 ± 310) were not statistically different from intestinal type of gastric cancers (183 ± 273) (p = 0.58). Neutrophils were also frequently observed in blood vessels of gastritis or gastric cancer tissues, which indicated that the source of neutrophils come from the circulating peripheral blood (Fig. 4B).

### Analysis on the relation of neutrophil infiltration and cell proliferation, E-cadherin expression

**Figure 5:**
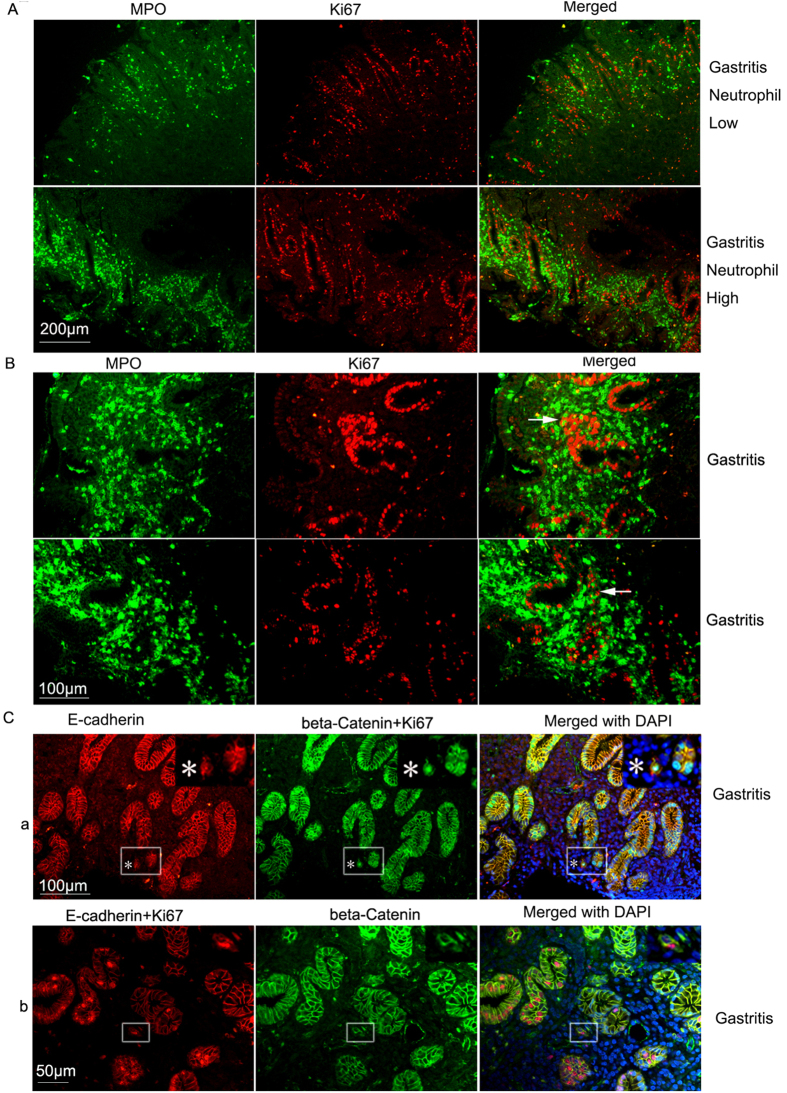
Neutrophils infiltration is associated with increased cell proliferation and also dissociated proliferating cells. (**A**) Neutrophil density is positively correlated with gastric epithelium cell proliferation in gastritis suggesting neutrophils might promote cell proliferation. Scale bar, 200 μm. (**B**) Neutrophils dissociate proliferating cells in gastritis. Arrows pointed to Ki67 positive cells intermingled with neutrophils. Scale bar: 100 μm (**C**) Detection of single proliferating gastric epithelium cell or small proliferating gastric epithelium cell clusters using triple antibody labeling. a), Single dissociated E-cadherin/beta-Catenin/Ki67 positive cells could be detected in gastritis tissues (Magnified images showed the single triple positive cell labeled with “*”. Scale bar, 100 μm; b), a two cell cluster with one Ki67 positive cell detected with another combination of E-cadherin/beta-Catenin/Ki67 triple antigen staining. Magnified images showed the E-cadherin/beta-Catenin/Ki67-positive 2 cell cluster. Scale bar, 50 μm.

To study the influence of neutrophil infiltration on gastric cancer development, we then analyzed the relation of neutrophil infiltration and gastric epithelium cell proliferation. As it was shown, the higher density of neutrophils was associated with higher density of proliferating gastric epithelium cells marked with Ki67 staining (Fig. 5A). Secondly, neutrophils seem to be dissociating proliferating gastric cells. Neutrophils are seen intermingled with disorganizing proliferating gastric epithelium cell clusters in gastritis tissues (Fig. 5B) and even single dissociated Ki67 positive epithelium cell could be observed. To prove that some of these proliferating dissociated cells are actually from the gastric epithelium, we did E-cadherin, beta-Catenin and Ki67 triple antigen co-staining but with two fluorescent channels. The experiment showed that a single E-cadherin (red label, cell membrane), beta-Catenin (green label, cell membrane), Ki67 (green label, nucleus) triple-positive proliferating gastric epithelium cell could be seen dissociated away in the gastritis tissues (Fig. 5C(a)). Although in theory beta-catenin could also be localized in the nucleus, but we did not observe predominant nuclear beta-catenin staining after staining hundreds of tissue samples. To avoid possible interference by nuclear beta-Catenin, we also did this three antigen labeling experiment using a different combination of 3 antibodies, with beta-Catenin labeled green (cell membrane), E-cadherin (cell membrane) and Ki67 (nucleus) both labeled red. Similar results were obtained (Fig. 5C(b)). E-cadherin, as a classical cell membrane adhesion molecule, was never found it to be in the nucleus in our experiments. These data indicated that the dissemination of small proliferating cell clusters into surrounding environments might be a common event during tissue repair under inflammatory conditions.

**Figure 6:**
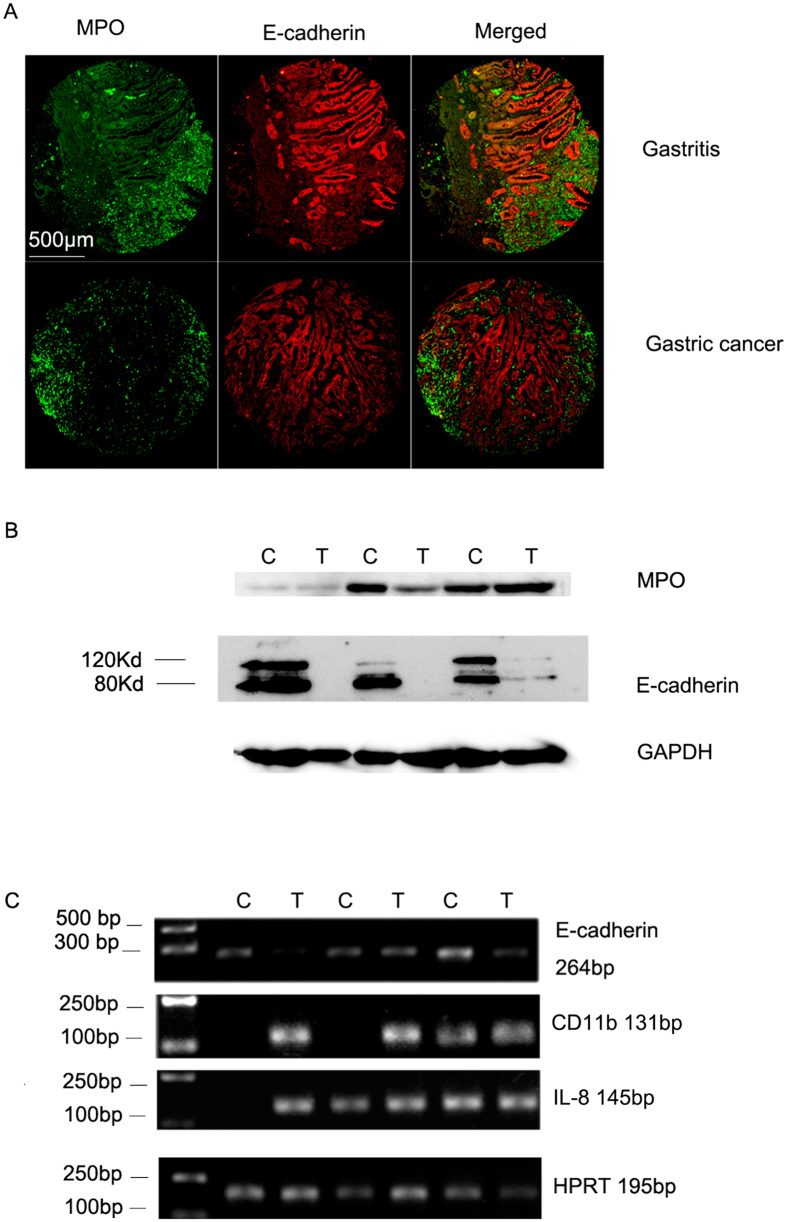
Neutrophil infiltration is associated with the downregulation of E-cadherin in both gastritis and gastric cancer tissues. (**A**) E-cadherin immunostaining was decreased in neutrophil infiltrated regions in both gastritis and gastric cancer tissues. Scale bar, 500 μm. (**B**) Relative MPO, E-cadherin protein expressions analyzed with western blots in tumor adjacent tissue controls (**C**) and tumor (gastric cancer) lysates (T). GAPDH is the loading control. Note that there is prominent E-cadherin degradation in gastric cancer tissues and gastric cancer adjacent tissues showing the full-length 120 Kd (top band) and the cleaved N-terminal domain of 80 Kd (bottom band). In addition, E-cadherin bands in gastric cancer tissues are much weaker than in the adjacent tissues. The original images for Fig. 6B could be seen in Supplementary Fig. 4. (**C**) E-cadherin is transcriptionally downregulated in gastric cancer tissues (T: tumor) comparing to adjacent tissues (C: controls) while the neutrophil marker CD11b and the neutrophil chemoattractant molecule IL-8 were both elevated in gastric cancer tissues comparing to adjacent tissues. The semi-quantitative PCR results of 3 paired samples were shown for E-cadherin, CD11b and IL-8. HPRT RT-PCR was served as the loading control. The original images for Fig. 6C could be seen in Supplementary Fig. 5.

Next, we analyzed the relation between neutrophil infiltration and E-cadherin expression in gastric cells and gastric cancer cells. It was found that neutrophil staining is inversely correlated with E-cadherin staining in both gastritis and gastric cancer tissues (Fig. 6A). We also analyzed the expression of MPO and E-cadherin protein levels in gastric cancer and cancer adjacent tissue control lysates by western blot (Fig. 6B). The original gel images for Fig. 6B could be found in Supplementary Fig. 4. E-cadherin protein on western blot showed characteristic two bands, a full length band of 120 Kd and an degraded band containing the E-cadherin ectodomain of 80 Kd. In cancer adjacent tissues, E-cadherin degradation is prominent, which correlates with the level of MPO protein expression. Western blot assay showed that E-cadherin was significantly downregulated in gastric cancer samples comparing adjacent tissues. Protein degradation of E-cadherin in gastric cancer tissues was also observed (Fig. 6B). However, in some cancer samples, E-cadherin expression totally disappeared, which might not be explained by protein degradation alone. The drastically diminished bands of E-cadherin in gastric cancer tissues suggested that E-cadherin might be also transcriptionally regulated. We then checked E-cadherin transcription by RT-PCR analysis. Semi-quantitative PCR results of E-cadherin and loading control HPRT showed that E-cadherin was indeed transcriptionally downregulated in gastric cancer tissues (Fig. 6C). The original gel images for Fig. 6C could be found in Supplementary Fig. 5. We also tried to correlate neutrophil markers with E-cadherin expression at transcription level. However, RT-PCR analysis of MPO failed after multiple tries. When searching the literature, we noticed that previous studies had already established that, comparing to abundant granule MPO protein in the differentiated neutrophils, MPO mRNA is no longer produced in differentiated neutrophils but abundantly expressed in neutrophil precursors and certain leukemia cells^36^,^37^,^38^. As suggested from the published study by Cowland JB *et al*.^36^, we decided to choose a late marker of neutrophils CD11b as the subject of transcription regulation analysis. RT-PCR showed that there is increasing CD11b transcription in gastric cancer tissues (Fig. 6C). The inverse relation of CD11b transcription and E-cadherin transcription indicates an negative correlation between neutrophil infiltration and E-cadherin expression. Since it had been suggested that neutrophil migration into gastritis tissues is regulated by IL-8^39^,^40^,^41^, we than did an IL-8 RT-PCR analysis. The data showed that there is also increasing IL-8 transcription in gastric cancer tissues comparing to cancer adjacent tissues (Fig. 6C). Quantitative RT-PCR showed that E-cadherin RNA expression is only around 44(±35)% of adjacent tissue controls and the difference is statistically significant (p = 0.02). Quantitative RT-PCR for CD11b and IL-8 also showed that they were upregulated. However, Ct values of CD11b and IL-8 were not obtained from some of the cancer adjacent tissues because of low abundance of the transcripts so that the exact degree of upregulation was not calculated.

### Analysis on the association of neutrophil infiltration and clinicopathological parameters of gastric cancer patients

For the contingency table analysis, we use 50 neutrophils as the cutoff value to divide 138 cancer samples into low neutrophil (57 samples) or high neutrophil count groups (81 samples) since there were roughly 50 neutrophils per sample in gastritis tissues. Surprisingly, we did not find significant association of neutrophil counts with patient ages (p = 0.84), sex (p = 0.79), tumor staging (p = 0.11), lymph node invasion (p = 0.56) or distal metastasis (p = 1) nor cancer types (p = 0.61) (Table 1).

## Discussion

Cancer development was previously describes as a “wounds never heal” process^42^. This study suggests neutrophils might be an important part in the “wounds never heal” model of tumorigenesis. The key finding in this study indicates that gastric cancer tissues are inflammation “hot spots” with abundant neutrophil infiltrations. The increasing neutrophil infiltrations from normal tissues to gastric cancer adjacent tissues and to gastric cancer tissues implicate that neutrophils may have a positive influence on gastric carcinogenesis or vice versa gastric carcinogenesis has a positive influence on neutrophil infiltration. Prior observations of increased neutrophil-lymphocyte ratio in the peripheral blood in cancer patients might be a reflex of the increased inflammation status in the cancer tissue environment^13^,^14^,^15^,^16^,^17^. The association between neutrophil infiltration with increased cell proliferation and E-cadherin downregulation suggested that neutrophils may somehow affect E-cadherin expression directly or indirectly and possibly affecting gastric carcinogenesis through E-cadherin since E-cadherin is linked to a number of important cell proliferation regulation pathways such as beta-Catenin and Hippo pathway^43^,^44^. This study also found that the neutrophil distribution patterns in intestinal type of gastric cancer and diffusive type of gastric cancer were different, suggesting the typing of gastric cancers is not only dependent on the characteristics of gastric cancer cells but also depending on the interactions between gastric cancer cells and immune cells such as neutrophils.

Several neutrophil enzymes were implicated in degrading the E-cadherin ectodomain, for example neutrophil elastase and MMP3^45^,^46^. Gaida, M. M. *et al*. did a detail study to show that neutrophil elastase specifically degrades E-cadherin in pancreatic cancers. MMP9 is another key neutrophil protease that might also modify E-cadherin, as suggested by a previous paper studying ovarian carcinoma^47^. However, our preliminary experiments using co-culture of E-cadherin expressing A549 cancer cells with HL-60 derived neutrophil-like cells did not confirm that a simple co-culture can reduce E-cadherin protein level effectively while trypsin protease can degrade E-cadherin ectodomain very effectively in a 2-mintue incubation (Supplementary Fig. 6). It is likely a more complicated process including multiple cell types and cell signaling is involved in E-cadherin downregulation *in vivo*. RT-PCR analysis in this study showed that E-cadherin is effectively transcriptionally downregulated in gastric cancer cells in addition to be modified by protein degradation. Further studies are required to find out the signaling and the exact roles of neutrophils in regulating E-cadherin at protein degradation and transcription regulation levels.

Data from this study indicates that neutrophil influx into gastric cancer tissues does not appear to be dependent on H. pylori infection of the adjacent tissues or EBV infection of gastric cancer tissues. An intriguing question is what drives the neutrophil infiltration into gastric cancer tissues. IL-8 has been implicated associating with neutrophil infiltration in gastritis tissues^39^,^40^,^41^. In this study, IL-8 and a neutrophil maker CD11b were upregulated in gastric cancer tissues concomitantly. IL-8 could be also an important molecule regulating the neutrophil influx into gastric cancer tissues. The exact reason of elevated IL-8 expression in gastric cancers is not clear. However, previous research showed that IL-8 is a target gene of ATM pathway in response to cancer-cell associated oxidative stress, which has a tumor promoting role in breast cancers^48^. IL-8 has also been shown to be secreted from oral squamous cell carcinoma cells, ovarian cancer cells and colon cancer cells, indicating the upregulation of IL-8 expression might be an universal phenomenon in cancer cells^49^,^50^,^51^. Further studies need to be carried out to investigate the mechanism of IL-8 upregulation in gastric cancers.

Earlier studies found that only 7.6% to 15.5% of gastric cancers are neutrophil-rich^22^,^23^, however, our finding is that nearly 60% of gastric cancers are neutrophil-rich. The difference could be due to the different standards to define neutrophil-rich samples in these studies. Since neutrophils have a short life-span of 5 to 6 days^6^, it is likely neutrophils often go through rapid cycles of life and death within the inflamed tissues, so that the presence of neutrophils might be still underestimated even with our analysis because the patient tissues were sampled at a particular time point but not followed continuously. We hope an effective way of *in vivo* real-time imaging of neutrophils could be established to localize and track the changes of cancer tissues dynamically. *In vivo* imaging of neutrophils might help to locate and monitor cancer mass and might also help with monitoring patients with other inflammatory conditions.

In summary, we studied the distribution of neutrophils in a large number of gastritis and gastric cancer tissues by immunohistochemisty. Increased neutrophil numbers was identified to be an important character of gastric cancer tissues. However, the difference of neutrophil numbers is not associated with gastric cancer patient age, sex, cancer stages, metastasis status or cancer types. Neutrophil infiltration is positively associated with cell proliferation and negatively associated with E-cadherin expression. Increased IL-8 expression was also observed in gastric cancer tissues, which could be a driver of neutrophil influx into gastric cancer tissues. The mechanism of transcriptional downregulation of E-cadherin during gastric inflammation and IL-8 upregulation in gastric cancer tissues require further studies.

## Materials and Methods

### Ethic Statement

The procedures relating to the acquisition of gastritis, cancer adjacent and gastric cancer frozen or paraffin tissue samples from patients were approved by the Ethics Committees of Guizhou Medical University Affiliated Hospitals and the First People’s Hospital of Shanghai Jiao Tong University and written informed consent was obtained from each subject. These tissue samples were taken from gastric biopsies or surgical tissues arising from medical procedures for diagnosis or treatments, which did not bring extra burden or pain to the patients. Guidelines from the Declaration of Helsinki were followed when the research was related to human subjects.

### Immunohistochemistry Analysis of Gastric Cancer Specimens and Tissue Array

Immunohistochemisty analysis of paraffin embedded specimens was carried out similarly as described^52^. Briefly, paraffin sections were deparaffinized with toluene and rehydrated with an inverse ethanol gradient following standard procedures. Sections were treated with 10 mM pH6.0 sodium citrate solutions with microwave at high power for 3 times of 4 minutes, with 5 minutes intervals. The sections were allowed to naturally cool down to room temperature. The sections were then blocked for one hour with 3% BSA in TBS with 0.1% Tween20. Further, the sections were incubated with primary antibodies diluted in the same blocking buffer for 2 hours at room temperature. The sections were then washed with TBS (0.1% Tween20) 5 times 5 minutes and then incubated with second antibodies diluted in the blocking buffer for 1 hour at room temperature. The sections were washed again with TBST, counterstained and mounted with PBS + 50%glycerol + DAPI (Sigma, St. Louis, MO, USA) mounting medium. All images were taken with a Leica DM6000B microscope (Leica Microsystems, Wetzlar, Germany) equipped with A4, L5 and N3 fluorescent filter cubes. The microscrope is equipped with 10X eyepiece, and 5X, 10X, 20X, 40X objective lens and 1.6X digital zoom capabilities. The images were taken using Leica Advanced Fluorescence software and analyzed with Image-Pro Plus software or examined manually by two researchers with the category of the samples blinded to the examiners. The mean of the two counting was used in the final statistical analysis. Only samples with over 50% tissues remaining on the array were counted for neutrophil numbers. To simplify the analysis, all neutrophils on the sections were counted without dividing the sections into sub regions. When comparing neutrophil numbers across different tissue arrays, neutrophil numbers were adjusted by the areas of the spotted arrays and section thickness. For the gastric cancer tissue array study, the first two gastric cancer and gastric intestinal metaplasia tissue arrays were purchased from a commercial tissue array vendor: Biomax, Rockville, MD, US with catalogue numbers ST1004 (Array 1, tissue spot diameter 1 mm, section thickness 5 μm) and IC00011b (Array 2, tissue spot diameter 1.5 mm, section thickness 5 μm). The third gastric cancer and adjacent tissue array were purchased from Shanghai Outdo Biotech Company with catalogue number HStm-Ade180CS-01 (Array 3, tissue spot diameter 1.5 mm, section thickness 4 μm).

### List of Antibodies for Immunohistochemistry

The following primary antibodies and secondary antibodies have been used: mouse monoclonal anti-Ki67 antibody (BD Biosciences, San Diego, CA, USA; 1:200); mouse monoclonal anti-CagA antibody (A-10) (1:200), rabbit polyclonal anti-CagA antibody (b-300) (1:200), rabbit anti-E-cadherin polyclonal antibody (H-108) (1:200), mouse anti-E-cadherin monoclonal antibody (G-10) (1:200) (All above antibodies were purchased from Santa Cruz Biotechnology Inc, Santa Cruz, California, USA); rabbit H. pylori polyclonal antibody (prediluted, ready to use), rabbit anti-Ki67 antibody (1:100) (Maixin Bio., Fuzhou, China); rabbit anti-MPO antibody (1:200), mouse anti-MPO antibody (1:200), rabbit anti-MMP9 antibody (1:200), rabbit anti-beta-Catenin antibody (1:200) (PTGLabs, Chicago, IL, USA); rabbit anti-Neutrophil elastase antibody (Abcam, Cambridge, MA,USA; 1:200); donkey anti-mouse Alexa Fluor 594 secondary antibody, donkey anti-rabbit Alexa Fluor 594 secondary antibody and donkey anti-rabbit Alexa Fluor 488 secondary antibody (Jackson ImmunoResearch, West Grove, PA, USA; 1:400).

### *In Situ* hybridization

EBV *in situ* hybridization assay were performed with a commercial kit following the manufacturer’s protocol with modifications to change from DAB detection to immunofluoresence detection (EBV *in situ* detection Kit, Catalogue Number: A020205, Triplex International Bioscience, Fuzhou, China). In brief, paraffin-embedded tissue sections are deparaffinized and treated with citrate antigen retrieval solutions similarly as above. The sections were then treated with proteinase K in PBS for 3 mins followed by ethanol dehydration. The dehydrated sections were then incubated with *in situ* hybridization buffers containing mixed digoxin-labeled EBV-specific EBER1 and EBER2 RNA probes in a hybridization chamber at 55 degree for 90 minutes in a humid box. The hybridization was continued by switching to 37 degree overnight incubation. On the second day, the hybridized sections were washed with PBS at 48 degree for 4 times of 5 minutes. The detection of the hybridization signals is through a mouse anti-digoxin primary antibody and a Donkey-anti-mouse Alexa Fluor 594 secondary antibody.

### Tissue Lysate Preparation and Western Blot Analysis

Gastric cancer and control tissues were homogenized by grinding in liquid nitrogen and sonicated on ice in High KCl lysis buffer (10 mM Tris-HCl, pH 8.0, 140 mM NaCl, 300 mM KCl, 1 mM EDTA, 0.5% Triton X-100 and 0.5% sodium deoxycholate) with complete protease inhibitor cocktail (Roche). Then the lysate was centrifuged at 13,000 g for 20 min at 4 °C. The supernatant was collected and quantified for protein concentration by BCA protein assay kit (Pierce, Rockford, IL, USA). Equivalent amounts of proteins were resolved by SDS-PAGE, and western blotting was performed with PVDF membrane transfer and detection with primary antibody incubation followed by secondary HRP-conjugated antibody incubation and ECL detection. The western blot gel image was obtained with an Minichemi 500 chemiluminescent imager (Sagecreation, Beijing, China). The antibodies that were used as follows: mouse anti-E-cadherin antibody (SantaCruz, 1:1000); rabbit anti-MPO antibody (PTG labs, 1:2000), mouse anti-GAPDH (PTG labs, 1:5000), goat anti-mouse HRP (PTG labs, 1:5000); goat anti-rabbit HRP (Signalway Antibody, College Park, MD, USA; 1:5000).

### RT-PCR

Total RNA of gastric cancer tissues and adjacent tissues was extracted using TRIZOL (Invitrogen) following the manufacturer’s protocol. cDNAs were generated from 1 μg of total RNA using reverse transcriptase with both random hexamer and oligo dT primers (Promega). Semi-quantitative RT-PCR was carried out in a 20 ul reaction volume with 10 ul 2 × Es Taq MasterMix (CWBio, Beijing, China), gene specific primers and cDNA templates. The PCR cycling condition is as the following: 94 degree 15 sec, 60 degree 30 sec, 68 degree 30 sec, for 32 cycles. 4 ul was taken from each PCR reaction tube and run on 1.2% agarose gel with ethidium bromide DNA dye. The agarose gel image was taken using a Tanon 2500 gel imager (Tanon, Shanghai, China). Quantitative real-time RT-PCR was performed using gene specific primers in a 20 μL reaction volume containing 10 μL 2 × SYBY Green Mix (GeneCopia, Rockville, MD, USA) and cDNA templates on the iQ5 (BioRad) machine. The quantitative PCR cycling condition was as the following: 94 degree 15 sec, 60.5 degree 30 sec for 40 cycles. The identity of PCR products was confirmed with melting curve analysis and also by agarose gel electrophoresis. The following primer sets were used in both regular PCR and quantitative PCR: HPRT (Forward primer: 5′-GGACTAATTATGGACAGGACTG-3′. Reverse primer: 5′-GCTCTTCAGTCTGATAAAATCTAC-3′)^53^; E-cadherin (Forward primer: 5′-ACGCTCGGCCTGAAGTGA-3. Reverse primer: 5′-ATTCGTTCAAGTAGTCATAGTCCTGG-3′); CD11b (Forward primer: 5′-TGGTACATCAAGACCTCGCATAAC-3′. Reverse primer: 5′-TCCACTTTGGTCTCCGTCTG-3′); IL-8 (Forward primer: 5′-TTGGCAGCCTTCCTGATTTC-3′. Reverse primer: 5′-CACTCTCAATCACTCTCAGTTCTTTG-3′). The relative abundance of transcripts was calculated from the differences of Ct values of E-cadherin RT-PCR against the differences of Ct values of loading control HPRT RT-PCR.

### Statistics Analysis

Statistics significance of the difference of the means was calculated with Mann-Whitney Test (for unpaired samples) or Wilcoxon Signed Rank Test (for paired samples). The statistical difference of the frequencies in contingency tables was analyzed with Pearson chi-square test or Fisher’s exact test (when the sample number is small). P < 0.05 is considered significant. The statistics was done using Excel with Megastat addin commands and IBM SPSS Statistics 19.

## Additional Information

**How to cite this article**: Fu, H. *et al*. Persisting and Increasing Neutrophil Infiltration Associates with Gastric Carcinogenesis and E-cadherin Downregulation. *Sci. Rep.* **6**, 29762; doi: 10.1038/srep29762 (2016).

## Supplementary Material

Supplementary Information

## Figures and Tables

**Table 1:** Relationship of neutrophil infiltration and clinicopathlogical parameters of gastric cancer patients.

**Pathological Variables**	**Number of each group**	**Neutrophil**		**p Value**
		**Low**	**High**	
**All cases**	138	57	81	
**Ages**				0.84
<60	64	27	37	
≧60	74	30	44	
**Gender**				0.79
Male	100	42	58	
Female	38	15	23	
**AJCC Staging**				0.11
I-II	86	31	55	
III-IV	52	26	26	
**Depth of Invasion**				0.46
T1-2	41	15	26	
T3-4	97	42	55	
				0.56
N0	67	26	41	
N1-3	71	31	40	
				1
M0	130	54	76	
M1	8	3	5	
**Cancer Type**				0.61
Diffusive	42	16	26	
Intestinal	96	41	55	

In total, 138 samples were analyzed. A neutrophil count less than 50 was defined as low neutrophil count while neutrophil number equal to or greater than 50 is considered as high neutrophil count. The statistics did not show significant association of neutrophil counts with patient ages, sex, tumor staging, lymph node or distal metastasis nor cancer types.

## References

[b1] Balkwill F. & Mantovani A. Inflammation and cancer: back to Virchow? Lancet 357, 539–545, doi: 10.1016/S0140-6736(00)04046-0 (2001). 11229684

[b2] Mantovani A., Allavena P., Sica A. & Balkwill F. Cancer-related inflammation. Nature 454, 436–444, doi: 10.1038/nature07205 (2008). 18650914

[b3] Feldman N., Rotter-Maskowitz A. & Okun E. DAMPs as mediators of sterile inflammation in aging-related pathologies. Ageing research reviews, doi: 10.1016/j.arr.2015.01.003 (2015). 25641058

[b4] Bei R., Masuelli L., Palumbo C., Modesti M. & Modesti A. A common repertoire of autoantibodies is shared by cancer and autoimmune disease patients: Inflammation in their induction and impact on tumor growth. Cancer letters 281, 8–23, doi: 10.1016/j.canlet.2008.11.009 (2009). 19091462

[b5] Mantovani A., Sozzani S., Locati M., Allavena P. & Sica A. Macrophage polarization: tumor-associated macrophages as a paradigm for polarized M2 mononuclear phagocytes. Trends in immunology 23, 549–555 (2002). 12401408 10.1016/s1471-4906(02)02302-5

[b6] Pillay J. . *In vivo* labeling with 2H2O reveals a human neutrophil lifespan of 5.4 days. Blood 116, 625–627, doi: 10.1182/blood-2010-01-259028 (2010). 20410504

[b7] Cools-Lartigue J., Spicer J., Najmeh S. & Ferri L. Neutrophil extracellular traps in cancer progression. Cellular and molecular life sciences: CMLS 71, 4179–4194, doi: 10.1007/s00018-014-1683-3 (2014). 25070012 PMC7096049

[b8] Fridlender Z. G. . Polarization of tumor-associated neutrophil phenotype by TGF-beta: “N1” versus “N2” TAN. Cancer cell 16, 183–194, doi: 10.1016/j.ccr.2009.06.017 (2009). 19732719 PMC2754404

[b9] Shau H. Y. & Kim A. Suppression of lymphokine-activated killer induction by neutrophils. J Immunol 141, 4395–4402 (1988). 3264311

[b10] Sagiv J. Y. . Phenotypic diversity and plasticity in circulating neutrophil subpopulations in cancer. Cell reports 10, 562–573, doi: 10.1016/j.celrep.2014.12.039 (2015). 25620698

[b11] Moses K. & Brandau S. Human neutrophils: Their role in cancer and relation to myeloid-derived suppressor cells. Seminars in immunology 28, 187–196, doi: 10.1016/j.smim.2016.03.018 (2016). 27067179

[b12] Sippel T. R. . Neutrophil degranulation and immunosuppression in patients with GBM: restoration of cellular immune function by targeting arginase I. Clinical cancer research: an official journal of the American Association for Cancer Research 17, 6992–7002, doi: 10.1158/1078-0432.CCR-11-1107 (2011). 21948231

[b13] Guthrie G. J. . The systemic inflammation-based neutrophil-lymphocyte ratio: experience in patients with cancer. Critical reviews in oncology/hematology 88, 218–230, doi: 10.1016/j.critrevonc.2013.03.010 (2013). 23602134

[b14] Peng B., Wang Y. H., Liu Y. M. & Ma L. X. Prognostic significance of the neutrophil to lymphocyte ratio in patients with non-small cell lung cancer: a systemic review and meta-analysis. International journal of clinical and experimental medicine 8, 3098–3106 (2015). 26064198 PMC4443032

[b15] Koh C. H. . Utility of pre-treatment neutrophil-lymphocyte ratio and platelet-lymphocyte ratio as prognostic factors in breast cancer. British journal of cancer 113, 150–158, doi: 10.1038/bjc.2015.183 (2015). 26022929 PMC4647546

[b16] Chen Z. Y. . Cytokine profile and prognostic significance of high neutrophil-lymphocyte ratio in colorectal cancer. British journal of cancer 112, 1088–1097, doi: 10.1038/bjc.2015.61 (2015). 25688736 PMC4366901

[b17] Shimada H. . High preoperative neutrophil-lymphocyte ratio predicts poor survival in patients with gastric cancer. Gastric cancer: official journal of the International Gastric Cancer Association and the Japanese Gastric Cancer Association 13, 170–176, doi: 10.1007/s10120-010-0554-3 (2010). 20820986

[b18] Marshall B. J. & Warren J. R. Unidentified curved bacilli in the stomach of patients with gastritis and peptic ulceration. Lancet 1, 1311–1315 (1984). 6145023 10.1016/s0140-6736(84)91816-6

[b19] Graham D. Y. Helicobacter pylori update: gastric cancer, reliable therapy, and possible benefits. Gastroenterology 148, 719–731 e713, doi: 10.1053/j.gastro.2015.01.040 (2015). 25655557 PMC4375058

[b20] Zhao J. J. . The prognostic value of tumor-infiltrating neutrophils in gastric adenocarcinoma after resection. PloS one 7, e33655, doi: 10.1371/journal.pone.0033655 (2012). 22442706 PMC3307751

[b21] Caruso R. A. . Prognostic value of intratumoral neutrophils in advanced gastric carcinoma in a high-risk area in northern Italy. Modern pathology: an official journal of the United States and Canadian Academy of Pathology, Inc 15, 831–837, doi: 10.1097/01.MP.0000020391.98998.6B (2002). 12181268

[b22] Rice A. J., Griffiths A. P., Martin I. G. & Dixon M. F. Gastric carcinoma with prominent neutrophil infiltration. Histopathology 37, 289–290 (2000). 10971710 10.1046/j.1365-2559.2000.01020-6.x

[b23] Ieni A. . Neutrophil-rich gastric carcinoma in the integrated cancer registry of eastern Sicily, Italy. Anticancer research 35, 487–492 (2015). 25550592

[b24] Yang G., Hu F. & Jia J. [Relation between infection of CagA-positive Helicobacter pylori and upper gastrointestinal diseases]. Zhonghua yi xue za zhi 81, 648–650 (2001). 11798940

[b25] He Y. . [Prevalence of cag A and vac A subtypes of Helicobacter pylori in Guangzhou]. Zhonghua nei ke za zhi 39, 818–820 (2000). 11798543

[b26] Chen X. J., Yan J. & Shen Y. F. Dominant cagA/vacA genotypes and coinfection frequency of H. pylori in peptic ulcer or chronic gastritis patients in Zhejiang Province and correlations among different genotypes, coinfection and severity of the diseases. Chinese medical journal 118, 460–467 (2005). 15788126

[b27] Li L., Kelly L. K., Ayub K., Graham D. Y. & Go M. F. Genotypes of Helicobacter pylori obtained from gastric ulcer patients taking or not taking NSAIDs. The American journal of gastroenterology 94, 1502–1507, doi: 10.1111/j.1572-0241.1999.01133.x (1999). 10364014

[b28] Yamaoka Y., Malaty H. M., Osato M. S. & Graham D. Y. Conservation of Helicobacter pylori genotypes in different ethnic groups in Houston, Texas. The Journal of infectious diseases 181, 2083–2086, doi: 10.1086/315486 (2000). 10837199

[b29] Crabtree J. E. . CagA/cytotoxic strains of Helicobacter pylori and interleukin-8 in gastric epithelial cell lines. Journal of clinical pathology 47, 945–950 (1994). 7962609 10.1136/jcp.47.10.945 PMC502181

[b30] Backert S. . Translocation of the Helicobacter pylori CagA protein in gastric epithelial cells by a type IV secretion apparatus. Cellular microbiology 2, 155–164 (2000). 11207572 10.1046/j.1462-5822.2000.00043.x

[b31] Odenbreit S. . Translocation of Helicobacter pylori CagA into gastric epithelial cells by type IV secretion. Science 287, 1497–1500 (2000). 10688800 10.1126/science.287.5457.1497

[b32] Stein M., Rappuoli R. & Covacci A. Tyrosine phosphorylation of the Helicobacter pylori CagA antigen after cag-driven host cell translocation. Proceedings of the National Academy of Sciences of the United States of America 97, 1263–1268 (2000). 10655519 10.1073/pnas.97.3.1263 PMC15590

[b33] Suzuki T. . Differential roles of interleukin-1beta and interleukin-8 in neutrophil transendothelial migration in patients with Helicobacter pylori infection. Scandinavian journal of gastroenterology 39, 313–321 (2004). 15125462 10.1080/00365520310008610

[b34] Nielsen H. & Andersen L. P. Chemotactic activity of Helicobacter pylori sonicate for human polymorphonuclear leucocytes and monocytes. Gut 33, 738–742 (1992). 1624151 10.1136/gut.33.6.738 PMC1379327

[b35] Shibata D. & Weiss L. M. Epstein-Barr virus-associated gastric adenocarcinoma. The American journal of pathology 140, 769–774 (1992). 1314023 PMC1886378

[b36] Cowland J. B. & Borregaard N. The individual regulation of granule protein mRNA levels during neutrophil maturation explains the heterogeneity of neutrophil granules. Journal of leukocyte biology 66, 989–995 (1999). 10614782 10.1002/jlb.66.6.989

[b37] Zaki S. R. . Human myeloperoxidase gene expression in acute leukemia. Blood 74, 2096–2102 (1989). 2553160

[b38] Tobler A. . Regulation of gene expression of myeloperoxidase during myeloid differentiation. Journal of cellular physiology 136, 215–225, doi: 10.1002/jcp.1041360203 (1988). 2842344

[b39] Uemura N. . Gastric corpus IL-8 concentration and neutrophil infiltration in duodenal ulcer patients. Alimentary pharmacology & therapeutics 11, 793–800 (1997). 9305491 10.1046/j.1365-2036.1997.00218.x

[b40] Ribeiro R. A., Flores C. A., Cunha F. Q. & Ferreira S. H. IL-8 causes *in vivo* neutrophil migration by a cell-dependent mechanism. Immunology 73, 472–477 (1991). 1916898 PMC1384579

[b41] Crabtree J. E. . Interleukin-8 expression in Helicobacter pylori infected, normal, and neoplastic gastroduodenal mucosa. Journal of clinical pathology 47, 61–66 (1994). 8132812 10.1136/jcp.47.1.61 PMC501759

[b42] Dvorak H. F. Tumors: wounds that do not heal. Similarities between tumor stroma generation and wound healing. The New England journal of medicine 315, 1650–1659, doi: 10.1056/NEJM198612253152606 (1986). 3537791

[b43] Van Aken E., De Wever O., Correia da Rocha A. S. & Mareel M. Defective E-cadherin/catenin complexes in human cancer. Virchows Archiv: an international journal of pathology 439, 725–751 (2001). 11787845 10.1007/s004280100516

[b44] Kim N. G., Koh E., Chen X. & Gumbiner B. M. E-cadherin mediates contact inhibition of proliferation through Hippo signaling-pathway components. Proceedings of the National Academy of Sciences of the United States of America 108, 11930–11935, doi: 10.1073/pnas.1103345108 (2011). 21730131 PMC3141988

[b45] Gaida M. M. . Polymorphonuclear neutrophils promote dyshesion of tumor cells and elastase-mediated degradation of E-cadherin in pancreatic tumors. European journal of immunology 42, 3369–3380, doi: 10.1002/eji.201242628 (2012). 23001948

[b46] Noe V. . Release of an invasion promoter E-cadherin fragment by matrilysin and stromelysin-1. Journal of cell science 114, 111–118 (2001). 11112695 10.1242/jcs.114.1.111

[b47] Symowicz J. . Engagement of collagen-binding integrins promotes matrix metalloproteinase-9-dependent E-cadherin ectodomain shedding in ovarian carcinoma cells. Cancer research 67, 2030–2039, doi: 10.1158/0008-5472.CAN-06-2808 (2007). 17332331

[b48] Chen W. T. . ATM regulation of IL-8 links oxidative stress to cancer cell migration and invasion. eLife 4, doi: 10.7554/eLife.07270 (2015). PMC4463759 26030852

[b49] Nishio Y., Gojoubori T., Kaneko Y., Shimizu N. & Asano M. Cancer cell-derived IL-8 induces monocytic THP1 cells to secrete IL-8 via the mitogen-activated protein kinase pathway. Tumour biology: the journal of the International Society for Oncodevelopmental Biology and Medicine 36, 9171–9177, doi: 10.1007/s13277-015-3641-6 (2015). 26088451

[b50] Singha B. . Proteasome inhibition increases recruitment of IkappaB kinase beta (IKKbeta), S536P-p65, and transcription factor EGR1 to interleukin-8 (IL-8) promoter, resulting in increased IL-8 production in ovarian cancer cells. The Journal of biological chemistry 289, 2687–2700, doi: 10.1074/jbc.M113.502641 (2014). 24337575 PMC3908402

[b51] Mei Q. . Methylation-induced loss of miR-484 in microsatellite-unstable colorectal cancer promotes both viability and IL-8 production via CD137L. The Journal of pathology 236, 165–174, doi: 10.1002/path.4525 (2015). 25727216

[b52] Fu H. L. . TET1 exerts its tumor suppressor function by interacting with p53-EZH2 pathway in gastric cancer. Journal of biomedical nanotechnology 10, 1217–1230 (2014). 24804542 10.1166/jbn.2014.1861

[b53] Valadan R. . Pseudogene-free amplification of HPRT1 in quantitative reverse transcriptase polymerase chain reaction. Analytical biochemistry 485, 46–48, doi: 10.1016/j.ab.2015.05.021 (2015). 26050630

