# Supplementary material for: Persisting and Increasing Neutrophil Infiltration Associates with Gastric Carcinogenesis and E-cadherin Downregulation
Source: Sci Rep. 2016 Jul 14;6:29762. doi: 10.1038/srep29762 (PMC4944193; doi:10.1038/srep29762)
Supplement: Supplementary Information [file srep29762-s1.doc]

**Supplementary Figures**

**Persisting and Increasing Neutrophil Infiltration Associates with Gastric Carcinogenesis and E-cadherin Downregulation**

Hualin Fu, Yue Ma, Meng Yang, Chunlei Zhang, Hai Huang, Ying Xia, Lungen Lu, Weilin Jin, Daxiang Cui


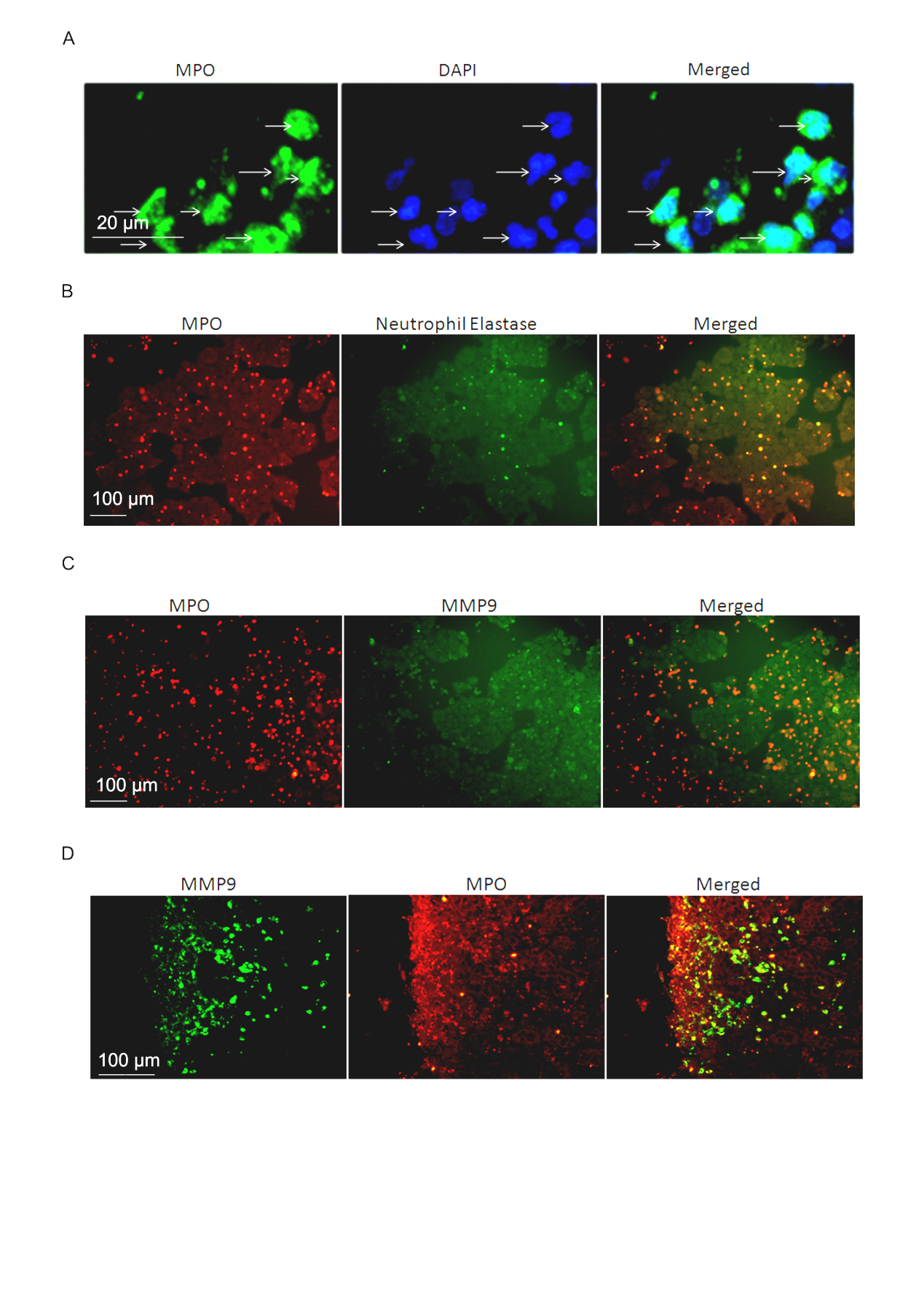


**Fig. 1: MPO stained neutrophils specifically.** A) MPO stained neutrophils showed characteristic multi-lobe nuclei. B) MPO co-stained with neutrophil elastase, a neutrophil marker, in a HL60-derived neutrophil-like cell and SC-7901 cancer cell co-culture model. C) MPO co-stained with MMP9, another neutrophil marker, in a HL60-derived neutrophil-like cell and SC-7901 cancer cell co-culture model. D) MPO co-stained with MMP9 on gastritis tissue sections.


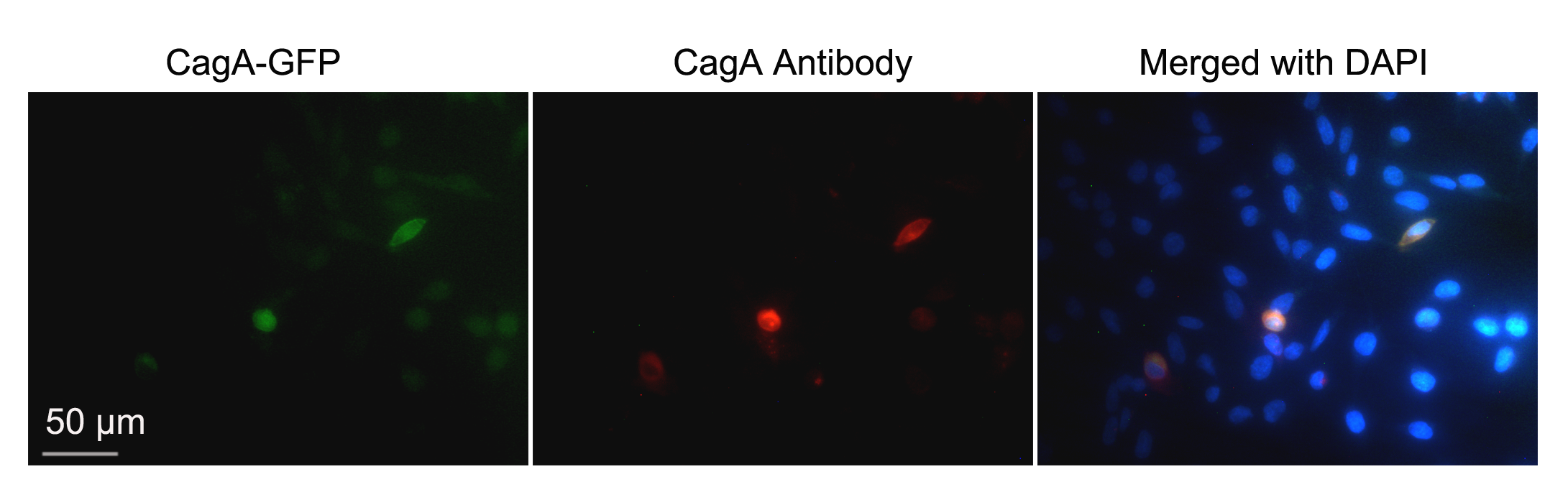


**Fig.2: Positive control experiment for CagA antibody.** CagA antibody is tested with CagA-GFP transfections with lipofactamine 2000 on MGC-803 gastric cancer cells. The co-localization of CagA-GFP (green) and CagA antibody (Santa Cruz Biotechnology Inc, Santa Cruz, California, USA) staining (red) is observed.


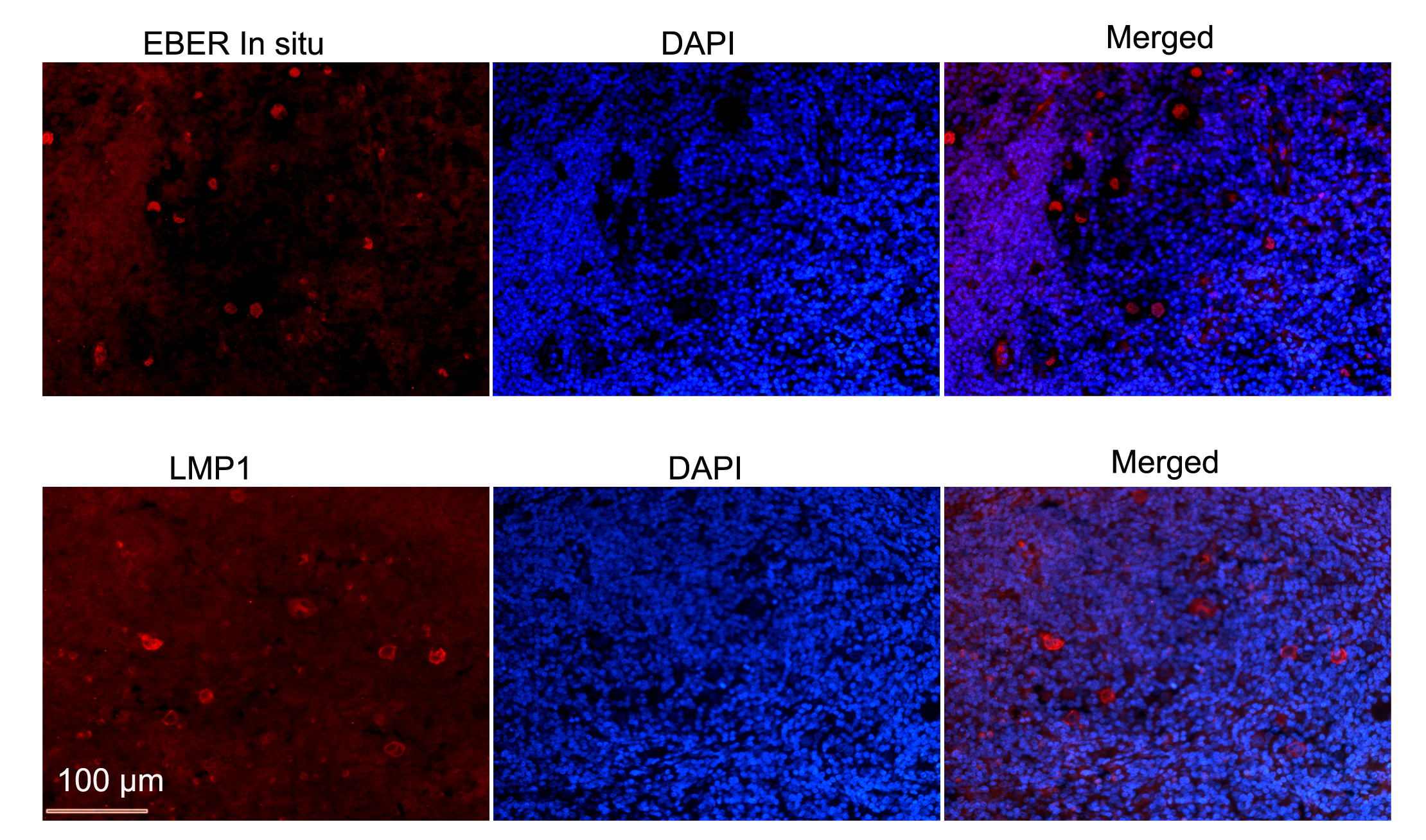


**Fig.3: Positive control experiment for EBV EBER in situ hybridization.** EBER in situ was performed on Hodgkin's lymphoma tissue sections (Maixin Bio., Fuzhou, China) (upper row) since Hodgkin's lymphoma is enriched for EBV infection, which could serves as the positive control for EBER in situ. EBER in situ correctly stained the Reed–Sternberg cells in the lymphoma tissues. Epstein–Barr virus latent membrane protein 1 (LMP1) antibody (Maixin Bio., Fuzhou, China) was also used to stain the lymphoma tissue sections （bottom row）. LMP1 antibody also stained the Reed–Sternberg cells clearly. However, on gastric cancer sections, only EBER in situ was able to identify EBV infections. LMP1 staining on gastric cancer sections was negative as previous studies have shown that LMP1 was scarcely expressed in EBV-infected gastric cancer cells.


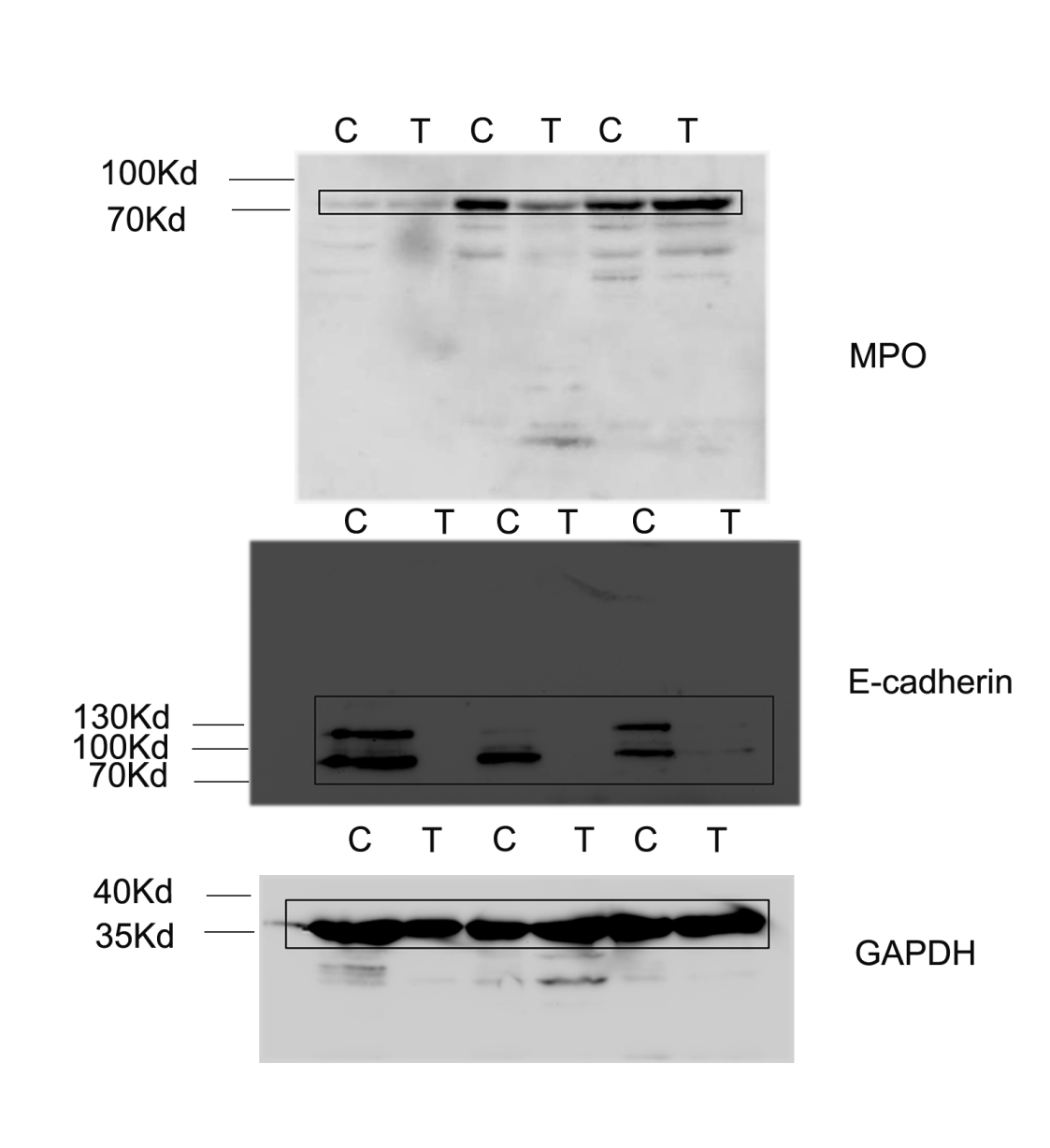


**Fig. 4: Relative MPO, E-cadherin protein expressions analyzed with western blots in tumor adjacent tissue controls (C) and tumor (gastric cancer) lysates (T).** GAPDH is the loading control. Note that there is prominent E-cadherin degradation in gastric cancer tissues and gastric cancer adjacent tissues showing the full-length 120Kd (top band) and the cleaved N-terminal domain of 80Kd (bottom band). The boxed regions showed the content appearing in the figures in the main text.


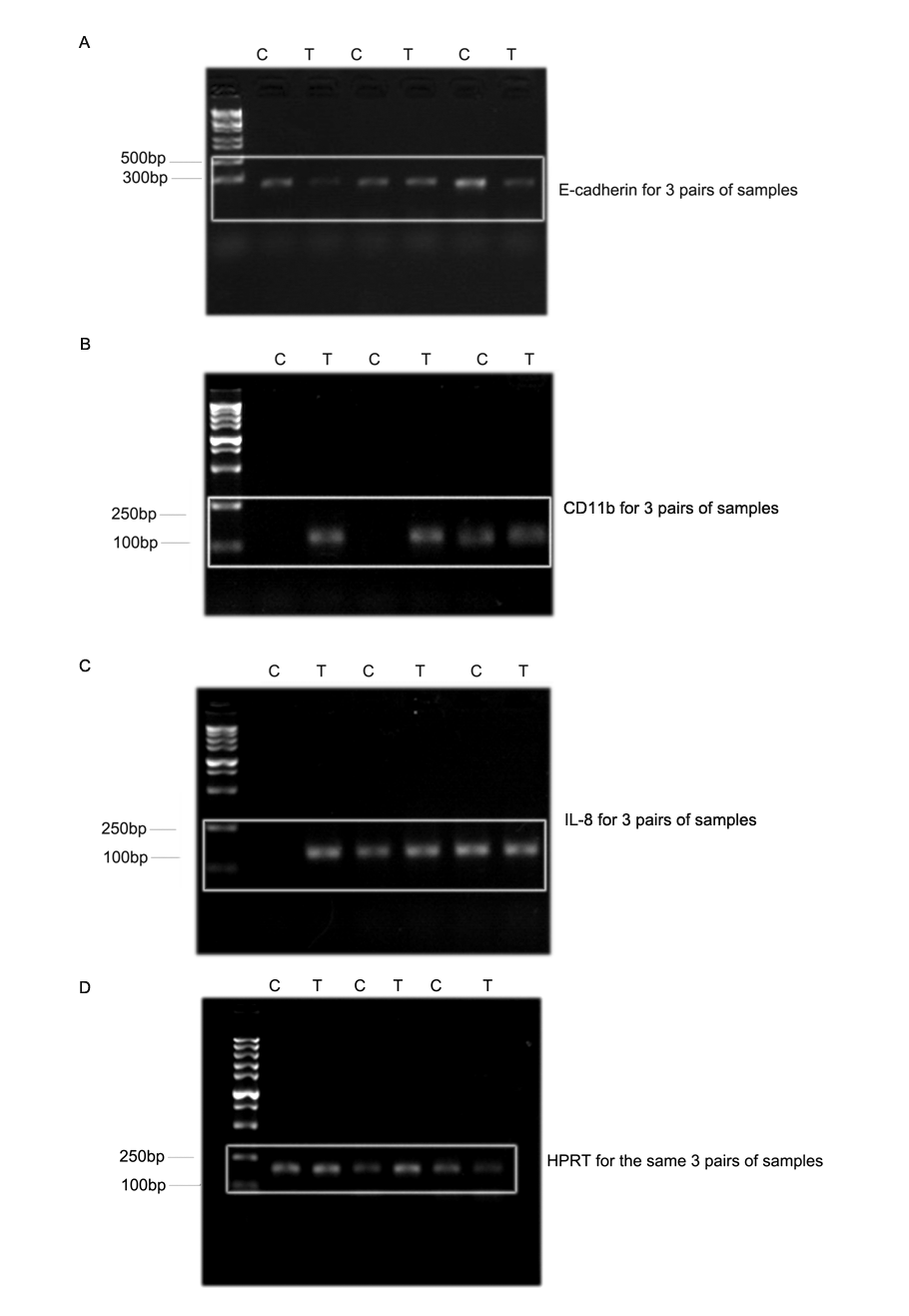


**Fig. 5: The original pictures of RT-PCR agrose gels to show that E-cadherin transcription was downregulated in gastric cancer tissues comparing to cancer adjacent tissues with increasing expression of the neutrophil marker CD11b** A) E-cadherin RT-PCR for 3 pairs of cancer tissues and adjacent tissue controls. PCR product for E-cadherin is 264bp. B) Neutrophil marker CD11b RT-PCR for 3 pairs of cancer tissues and adjacent tissue controls. PCR product for CD11b is 131bp. C) Neutrophil chemoattractant IL-8 RT-PCR for 3 pairs of cancer tissues and adjacent tissue controls. PCR product for IL-8 is 145bp. D) HPRT RT-PCR for the same 3 pairs of cancer tissues and adjacent tissue controls. PCR product for HPRT is 195bp. Note: DNA molecular weight marker in A is different from the MW marker in B, C and D. C: adjacent tissue controls. T: tumors (cancers). The boxed regions showed the content appearing in the figures in the main text.


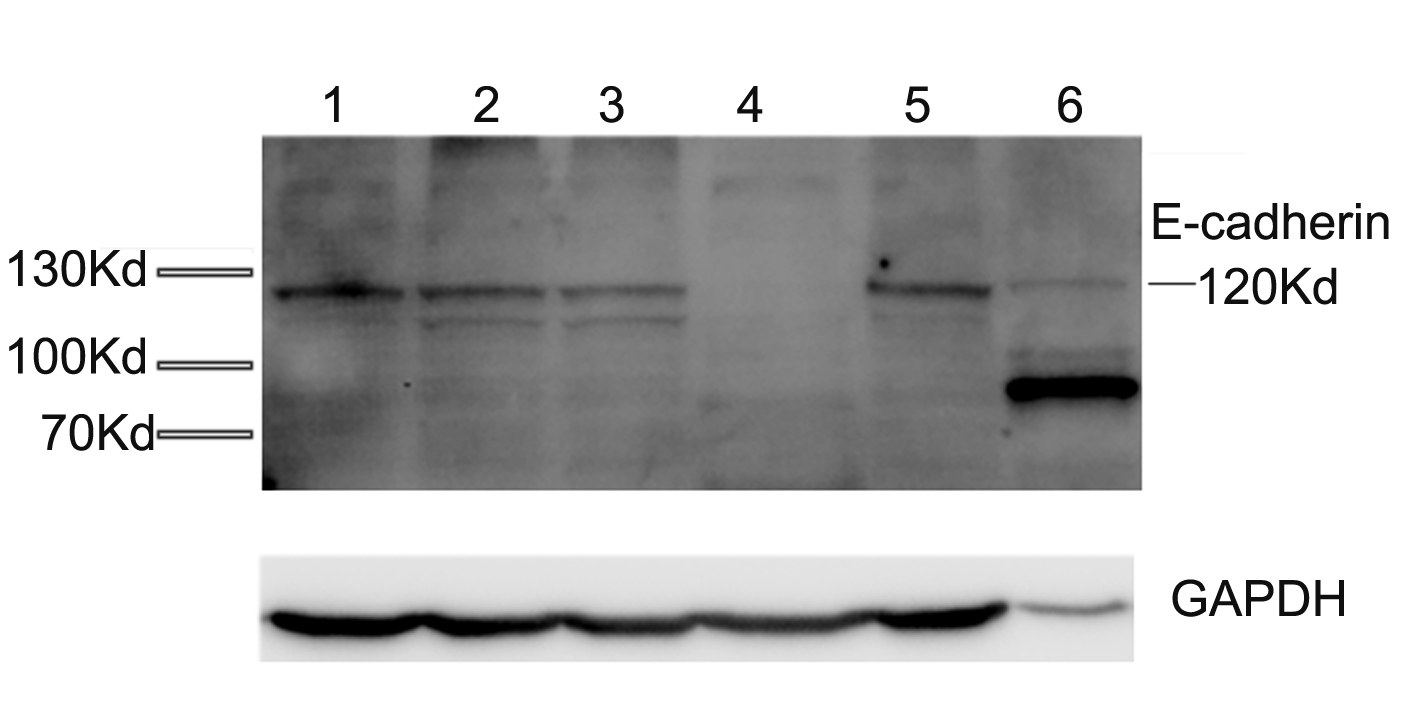


**Fig. 6： Simple co-culture with HL-60 derived neutrophil-like cells did not reduce E-cadherin protein of A549 cancer cells.** 1, Cell lysate from A549 cells. 2, Cell lysate from A549 cells that co-cultured with LPS (10ng/ml) pretreated 1.25% DMSO differentiated neutrophil-like cells derived from HL-60 cells for 2hrs in no-serum medium. 3. Cell lysate from A549 cells co-cultured with 1.25% DMSO differentiated neutrophil-like cells derived from HL-60 cells at the presence of LPS for 2hrs in no-serum medium. 4, Cell lysate from A549 cells that were washed with PBS and then treated with trypsin-EDTA for 2 minutes. 5, Cell lysate from A549 cells co-cultured with 1.25% DMSO differentiated neutrophil-like cells derived from HL-60 cells at the presence of LPS for 15 minutes in PBS. 6, Cell lysate from a gastric cancer tissue sample. Note, since we did not have enough cell lysate from the cancer tissue, the cancer tissue sample (No. 6 sample) was not loaded the same amount as the cell culture lysates.
